# Location-Specific Responses to Thermal Stress in Larvae of the Reef-Building Coral *Montastraea faveolata*

**DOI:** 10.1371/journal.pone.0011221

**Published:** 2010-06-23

**Authors:** Nicholas R. Polato, Christian R. Voolstra, Julia Schnetzer, Michael K. DeSalvo, Carly J. Randall, Alina M. Szmant, Mónica Medina, Iliana B. Baums

**Affiliations:** 1 Department of Biology, Pennsylvania State University, University Park, Pennsylvania, United States of America; 2 Red Sea Research Center, King Abdullah University of Science and Technology, Thuwal, Saudi Arabia; 3 Max Planck Institute for Marine Microbiology, Bremen, Germany; 4 School of Natural Sciences, University of California Merced, Merced, California, United States of America; 5 Center for Marine Sciences, University of North Carolina Wilmington, Wilmington, North Carolina, United States of America; University of Sydney, Australia

## Abstract

**Background:**

The potential to adapt to a changing climate depends in part upon the standing genetic variation present in wild populations. In corals, the dispersive larval phase is particularly vulnerable to the effects of environmental stress. Larval survival and response to stress during dispersal and settlement will play a key role in the persistence of coral populations.

**Methodology/Principal Findings:**

To test the hypothesis that larval transcription profiles reflect location-specific responses to thermal stress, symbiont-free gametes from three to four colonies of the scleractinian coral *Montastraea faveolata* were collected from Florida and Mexico, fertilized, and raised under mean and elevated (up 1 to 2°C above summer mean) temperatures. These locations have been shown to exchange larvae frequently enough to prevent significant differentiation of neutral loci. Differences among 1,310 unigenes were simultaneously characterized using custom cDNA microarrays, allowing investigation of gene expression patterns among larvae generated from wild populations under stress. Results show both conserved and location-specific variation in key processes including apoptosis, cell structuring, adhesion and development, energy and protein metabolism, and response to stress, in embryos of a reef-building coral.

**Conclusions/Significance:**

These results provide first insights into location-specific variation in gene expression in the face of gene flow, and support the hypothesis that coral host genomes may house adaptive potential needed to deal with changing environmental conditions.

## Introduction

Coral populations are declining worldwide due to rising sea surface temperatures (SSTs), overfishing, coastal development, and pollution [1]. This population reduction has been exacerbated in Caribbean reefs by declining juvenile recruitment, reduced growth, and increased mortality [2], [3], [4]. Successful sexual reproduction is necessary for recovery and persistence of these ecosystems because it maintains genetic diversity within, and connectivity among, benthic adult populations. Because larvae have limited energetic reserves, the impact of temperature stress on these early stages of development may differ significantly from that seen in adult coral colonies [5], [6]. As such, changing temperature regimes are likely to have profound effects on larval-mediated ecological processes including dispersal, connectivity, and population dynamics of reef-building corals [5], [6], [7], [8]. These effects will ultimately have consequences on coral populations' ability to adapt to a changing climate.

Determining the effects of thermal stress on corals has been a major focus of research since the observation that elevated temperatures, such as those caused by El Niño events, can result in bleaching and subsequent high mortality on affected reefs [9], [10]. A complex interplay among the animal host, its symbiotic algae [11], [12], [13], and the microbial community inhabiting the mucus layer [14], [15], [16] enable the holobiont (the coral animal with its algal and bacterial symbionts) to respond to changing conditions. The ability to respond and ultimately adapt to thermal stress will be vital for the continued survival of corals in the face of global climate change [1], [17].

There has been much debate regarding the adaptive potential of coral hosts. Because it is widely accepted that corals exist near their thermal maxima throughout much of their range [18], their ability to adapt to changing climate conditions has been questioned [1]. However, local adaptation, once thought to be minimal due to long distance gene flow among marine populations, has since been reconsidered in light of small scale population structure in multiple coral species [7]. Recent models of coral survival have incorporated parameters that consider adaptive potential (such as increased thermotolerance), resulting in considerably different outcomes depending on the strength of the adaptive response [19], [20], [21]. Past studies have explored the mechanisms by which corals may acclimate and/or adapt to elevated temperatures [22], [23], but empirical tests of coral performance in response to thermal stress have been limited to studies of bleaching and mortality in adult corals [18], [24], [25], [26], until recently [27], [28], [29].

Each member of the holobiont contributes to the fitness of a coral colony, and distinguishing among the fitness contributions of each is vital to our understanding of the adaptive potential inherent to coral populations. The most direct method for investigating the host response in isolation is to work with coral larvae, as many species do not take up algal, and possibly microbial, symbionts until late in their larval development. Thus, utilizing aposymbiotic larvae (without symbionts) allows for isolation of symbiont-free genetic material, so any indicators of stress can be confidently assigned to the host animal.

Effects of environmental stress on coral larvae include altered developmental rates, abnormal morphologies, changes in settlement behavior and reduced survival [30], [31], [32], [33], [34], [35]. Measurements on larvae of the Elkhorn Coral (*Acropora palmata*) show that increases in temperature of only 2 degrees can decrease survivorship, accelerate developmental rates, and increase swimming speed, suggesting a host of accompanying physiological and metabolic changes with consequences for important ecological processes including recruitment, dispersal and connectivity [35, Baums et al., unpublished data]. While larvae are expected to react to environmental stress differently than adults, an understanding of the molecular stress response in this vulnerable and critically important life stage is warranted. Additionally, identification of differentially expressed genes in larvae may offer a first clue to important stress tolerance genes in adults.

Larval response to stress has previously been measured using the limited amount of phenotypic characters (morphological, behavioral, survival) currently available for coral larvae. These are restricted to the few clearly identifiable embryonic developmental stages, and obvious pathological malformations that are observed at high temperatures [31], [33]. By treating gene expression levels as molecular phenotypes, microarray technology greatly increases the number of phenotypic traits available for assessing the effect of stress on corals because we are able to survey the expression of thousands of transcripts simultaneously [27], [28], [36], [37]. Additionally, with ever advancing functional annotation of genes and genomes in many organisms, results can be interpreted to better understand the mechanisms underlying key processes such as the thermal stress response.

Examination of the molecular response of cnidarians to high temperatures has revealed a wide range of variation in molecular phenotypes. These include changes in expression levels of the ubiquitous families of heat shock proteins (HSPs) [38], and multiple genes involved in defense from oxygen radicals [36], [39], [40], [41]. Gene expression studies on larval and juvenile corals have identified differential regulation of genes involved in thermal and oxidative stress response, apoptosis, and cytoskeletal structuring, among others [27], [28], [29]. Assessing gene expression levels can reveal stress prior to the onset of obvious pathologies and gives an immediate snapshot of the organism's health faster than many traditional metrics such as changes in growth rate, mortality, or fertility [42], [43].

In this study we compare gene expression profiles of embryos of the common Caribbean coral *Montastraea faveolata*. Adult colonies of this species are hermaphroditic and reproduce annually during the late summer when seawater temperatures are maximal, by spawning gametes into the water column where fertilization occurs [44]. After fertilization, embryos develop into larvae and drift with the currents for up to two weeks before settling onto the benthos where they metamorphose into a primary polyp. Embryos of *M. faveolata* raised from spawn collected from Florida and Mexico were reared under elevated temperatures (1 to 2°C over local summer means) and less stressful control temperatures based on the year round means, to test the hypothesis that transcription profiles reflect location-specific responses to thermal stress. We expected that the thermal stress response would include differential expression of genes for previously identified markers of stress, such as those coding for HSPs, and oxidative stress proteins. Additionally, we anticipated differential expression of genes involved in cell structure and development that may play a role in the irregular morphology observed in embryos reared at high temperatures [31], [35].

The results presented here expand our understanding of the effect of temperature stress on gene expression during embryonic development in *M. faveolata* as described in Voolstra *et al.* [27], by extending the analysis from 12 to 24 hours of development. This enables a more continuous view of embryonic development over the first two days because the 48 hour samples from Mexico used here are the same as those used in Voolstra *et al.* [27].

## Materials and Methods

**Figure 1:**
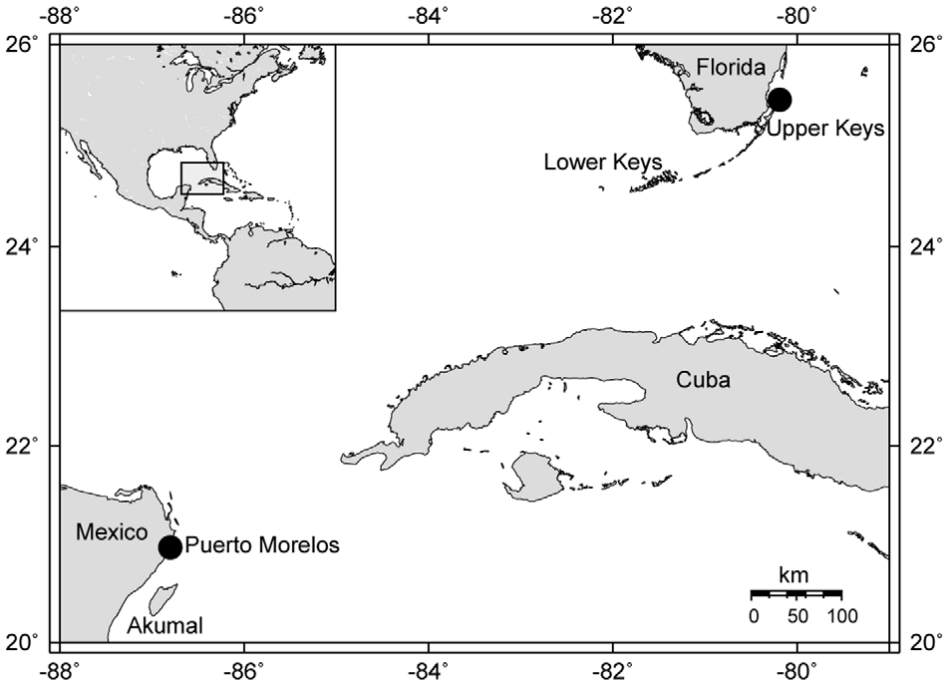
Map of the western Atlantic and northern Caribbean with study locations indicated by black circles.

Gametes from multiple parent colonies of *M. faveolata* were collected during mass spawning events at two locations: Puerto Morelos, Mexico (20°52′28.77″N, 86°51′04.53″W) and Key Largo, Florida (25°6′42.66″N, 80°18′18.72″W) (Fig. 1). Parent genotypes were reconstructed using 5 previously published polymorphic microsatellite loci [45] and estimates of allelic diversity across all five loci were similar in both sample populations (Mexico 14 alleles, Florida 19 alleles). This corresponded to three to four parental genotypes contributing to the gene pool of each batch and captured around 25% of the local allelic diversity (unpublished data). STRUCTURE analysis of five polymorphic microsatellite loci (unpublished) and previous population genetic analyses [46] showed that these populations exchange larvae frequently enough to prevent significant differentiation.

**Figure 2:**
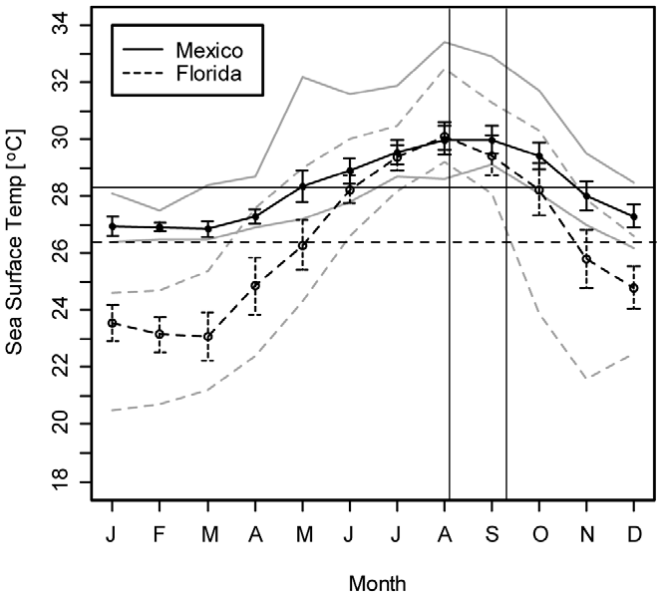
Mean monthly sea surface temperatures from 2005 to 2008 for the two study locations (Mexico and Florida). Horizontal lines represent annual means. Area within the vertical lines delineates the spawning season for *M. faveolata*. Error bars indicate ±1 standard deviation. Grey lines represent monthly maxima and minima. Data from NOAA weather buoys MLRF1 and 42056.

Sperm and eggs from multiple parents were pooled and incubated for one hour to allow for fertilization, resulting in one batch with multiple parents for each location. Fertilized eggs were washed and transferred to temperature-controlled aquaria. In Florida, larvae were raised in 1 L plastic containers with mesh sides to allow for water exchange, suspended in 6 separate 45 L polycarbonate bins containing filtered sea water (3 at each treatment temperature). Water was circulated with an aquarium pump and changed daily with filtered sea water preheated to the target temperature. Target temperatures were maintained within ±0.6°C with aquarium heaters and chiller units, and were monitored with HOBO temperature data loggers (Onset Computer Corp., MA) in each bin. The temperature exposure system used for the Mexico embryos maintained target temperatures within ±0.2°C and is described in detail in [35]. Briefly, embryos were cultured in three 500 mL plastic containers suspended in each of two temperature controlled 12 L polycarbonate bins at treatment and control temperatures. Water in the containers was changed twice daily with water preheated to the desired temperature by siphoning out the old sea water through a sieve made of PVC pipe and 120 um mesh. This removed many of the smaller particles and dying embryos that were smaller than the mesh. Respective annual and summer mean temperatures from 2005 to 2008, calculated by averaging monthly means of hourly data, are 26.4°C and 29.3°C in Florida and 28.3°C and 29.5°C in Mexico. Control temperatures were near the annual mean for both locations (27°C Florida and 27.5°C Mexico; “mean”). High treatment temperatures were 1 to 2°C above the summer means (30°C Florida and 31.5°C Mexico; “high”) (Fig. 2).

Embryos for the microarray analysis were preserved in RNAlater (Ambion, TX) after approximately one full day (22 to 24 hours; “24 hours”) and two full days (46 to 48 hours; “48 hours”) of development and stored at −80°C until RNA extraction was performed. Each sample consisted of ca. 1500 genetically diverse embryos. In Florida, one sample was taken from each of the three replicate bins at each time and temperature (with the exception of the 48 hour sample at the high temperature where there were only enough embryos remaining in two of the three replicates). Biological replicates from Florida were used to interrogate a single microarray slide each. Sampling in Mexico was as described in [27], where embryos from each of the three containers in the two replicate bins at a given temperature were combined into a single composite biological replicate at both time points. Three technical replicate arrays were run using RNA from each composite biological sample from both temperatures and time points.

Changes in water temperature can modify embryonic developmental rates and thereby alter gene expression patterns. To verify that expression differences among temperatures for a given sampling time were not simply due to developmental differences among samples, embryos from both treatment temperatures were preserved in formalin or glutaraldehyde after 22 and 46 hours in Florida, and 21.5, 28, and 50.5 hours in Mexico and viewed under a dissecting microscope for classification by developmental stage (Table 1). Visual assignment to any developmental stage is approximate given that classification is based solely on external morphology. By the time of blastula formation, the embryos are too opaque to distinguish gradual internal changes associated with gastrulation and development into planulae. Embryos reach a blastula-like stage by ca. 6–8 hours after fertilization, and take ca. 20 more hours to transition from blastulae to full gastrulae (invagination fully accomplished). It takes a further 40 hours to reach the planula stage [47]. Since fertilization occurred over a one hour period, there was often more than one identifiable stage present at a given time point during early cleavage and development, but we were not able to distinguish between subtle differences during later embryonic stages.

**Table 1:** Percentages of *M. faveolata* embryos in various stages of development at control and treatment temperatures from Mexico and Florida.

Location	Age [hours]	Temperature [°C]	Total Embryos	Irregular Embryos [%]	Normal Embryos as Blastulae [%]	Normal Embryos as Invaginated Blastulae [%]	Normal Embryos as Gastrulae [%]	Normal Embryos as Planulae [%]
Florida	22	27	141	6	4	96	0	0
	22	30	57	7	2	98	0	0
	46	27	66	0	0	3	95	2
	46	30	34	50	0	0	100	0
Mexico	21.5	27.5	100	4	6	94	0	0
	21.5	31.5	101	8	1	99	0	0
	28	27.5	100	9	0	100	0	0
	28	31.5	100	20	0	0	100	0
	50.5	27.5	100	11	0	0	99	1
	50.5	31.5	101	4	0	0	100	0

The percentage of irregular embryos is taken from the total number observed. Age is given in hours after fertilization. The percentage of normal embryos was calculated after subtracting the irregular embryos which could not be scored for developmental stage. Mexico data for 50.5 hours are modified from Voolstra *et al.* [27].

RNA was extracted from approximately 1,500 embryos from each sample and was used to interrogate a 2,620 feature (1,310 double spotted unigenes) custom array [36], [48]. Microarray protocols were those of the Center for Advanced Technology at the University of California, San Francisco (http://cat.ucsf.edu/). Total RNA was extracted using the RNeasy Mini Kit (QIAGEN, CA). Concentration and quality of RNA extracts were quantified on a NanoDrop ND-1000 spectrophotometer, and an Agilent 2100 Bioanalyzer. To prepare RNA for microarray hybridization 1 µg of total RNA was amplified with the MessageAmp II aRNA Kit (Ambion, TX). Bias associated with this process is considered negligible [49]. To prime the reverse transcription (RT) reaction 3 µg of aRNA was incubated with 2 µL of 5 µg/µL random nonamers for 10 minutes at 70°C. RT was carried out for 2 hours at 50°C using a master mix with a 4∶1 ratio of aminoallyl-dUTP to TTP. Products of the RT reaction were hydrolyzed by incubating the cDNA in 10 µL of 0.5M EDTA and 10 µL of 1M NaOH for 15 minutes at 65°C. Following hydrolysis, RT products were purified using MinElute columns (Qiagen, CA), and cDNA synthesis was checked on a NanoDrop spectrophotometer. Dye coupling reactions were performed using Cy3 and Cy5 dyes (GE Healthcare, PA) diluted in 18 µL of dimethyl sulphoxide. The coupling reactions were run in the dark for 2 hours at room temperature. A final cleanup was performed using the MinElute Cleanup Kit, and dye coupling was confirmed on a NanoDrop spectrophotometer. Before hybridization microarray slides were post-processed by UV crosslinking at 60 mJ; “shampooing” with 3×SSC and 0.2% SDS at 65°C; blocking with 5.5g succinic anhydride in 335 mL 1-methyl-2-pyrrilildinone and 15ml sodium borate; and drying via centrifugation. Dye coupled cDNAs were then mixed together in a hybridization buffer consisting of 0.25% SDS, 25 mM HEPES, and 3×SSC. Hybridization mixtures were boiled at 99°C for 2 minutes then allowed to cool at room temperature for 5 minutes. Cooled hybridization mixtures were pipetted under an mSeries Lifterslip (Erie Scientific, NH) and slides were incubated overnight at 63°C in a custom hybridization chamber. Hybridized microarray slides were then washed twice in 0.6×SSC and 0.01% SDS, rinsed in 0.06×SSC, and dried via centrifugation. Microarrays were scanned using an Axon 4000B slide scanner (MDS, CA).

As cluster analysis was the major goal of this study the microarray experiment followed a reference design, where all samples were hybridized against a common reference sample. The pooled reference sample consisted of equal amounts of RNA from all Mexico samples only, due to the fact that the Mexico samples were processed in advance of the Florida samples. Reference samples were labeled with Cy3, and temperature treatment samples were labeled with Cy5. Because multiple factors were targeted (time, temperature, and location), and the reference sample was of no biological interest, dye swaps were not performed [50].Three replicates were run for each temperature treatment (except for the 48 hour high temperature sample from Florida). Data from the microarrays are available from the Gene Expression Omnibus Database (NCBI; GSE19998).

Spot intensities were extracted and background was subtracted using GenePix Pro 6.0. GPR files were read into the Bioconductor package LIMMA for further analysis in R [51], [52]. Normalization was performed using print-tip specific LOWESS to normalize within arrays and the quantile normalization method to normalize between arrays [53]. LIMMA uses linear regression models to incorporate the power of replicated experimental design into gene expression analysis. P-values were adjusted using an empirical Bayes shrinkage of standard error, and false discovery rate was corrected using the method of Benjamini and Hochberg [54]. Finally, a log fold change cutoff of 1.5 and a p-value threshold of 0.05 were used to filter significant results.

Significant DEGs were categorized based on cellular function according to GO (Gene Ontology) and KEGG data (Kyoto Encyclopedia of Genes and Genomes). Clone sequences are available at http://sequoia.ucmerced.edu/SymBioSys/index.php. Lists of differentially expressed genes (DEGs) were generated and the overlap among the lists was visualized in Venn diagrams constructed with Limma. Hierarchical clustering of transcriptome profiles from model fitted results and computation of associated p-values via bootstrap resampling was performed using the R package pvclust [55]. The tree was visualized with FigTree 1.2.3 (http://tree.bio.ed.ac.uk/software/figtree/).

Unsupervised dimension reduction via principle components analysis (PCA) was also carried out in R. PCA was used to identify basic patterns among the highly dimensional gene expression profiles. Briefly, PCA transforms possibly correlated variables into a smaller number of uncorrelated variables called principle components. Expression profile data from each treatment was transformed to extract the principle components responsible for the greatest amount of variance. The principle components were then plotted in order of their contribution to the overall variance.

## Results

### Larval Development Patterns

In Mexico, developmental samples were collected just before (21.5 hours) and just after (28 and 50.5 hours) the microarray samples (24 and 48 hours). At 21.5 hours of development the embryos were predominantly blastulae in the process of gastrulation. By 28 hours, all of the embryos at the high temperature were in the gastrula stage, while those at lower temperature still had large invaginations. These data suggest that the embryos at 24 hours were in the process of gastrulation, and that some proportion of the embryos at the high temperature were more developmentally advanced than those at the lower temperature (Table 1). By 50.5 hours, embryos at both temperatures were in the late gastrula stage, but had not begun to swim or elongate into planulae. Florida embryos collected at 22 hours were also predominantly in the process of gastrulation. By 46 hours they were fully formed gastrulae, indicating that embryos used for the microarray experiment from a single time point were predominantly at the same developmental stage across temperature treatments and locations.

Interestingly, cultures reared at the higher temperature in Mexico and Florida included numerous misshapen embryos, confirming similar observations in other studies of coral development at elevated temperatures [30], [33], [35]. In Florida, embryos raised at the higher temperature were most strongly affected, where 21% and 50% (22 and 46 hours respectively) were malformed.

### Gene Expression Patterns

#### Hierarchical gene clustering

**Figure 3:**
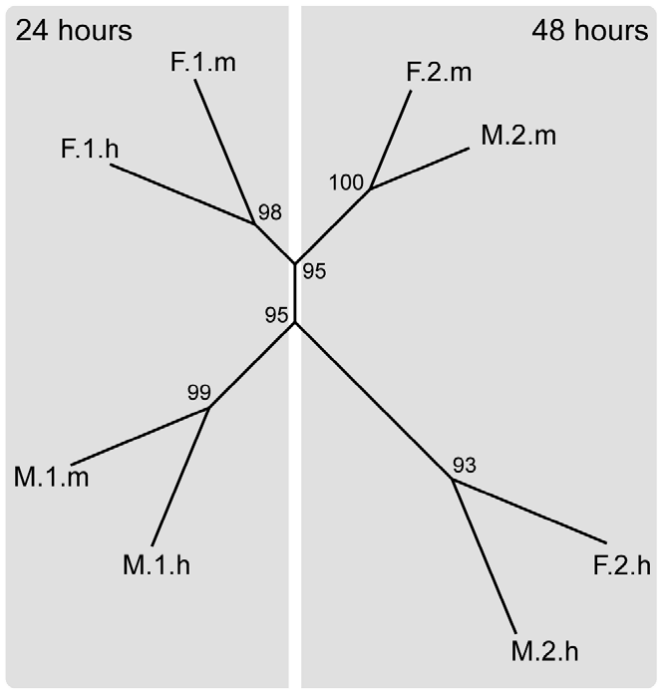
Radial tree of hierarchically clustered gene expression profiles for *M. faveolata* embryos collected from Florida and Mexico after 24 and 48 hours of development. All three factors of time, location, and temperature played a role in shaping gene expression profiles. Note that clustering of day one samples was according to location (M and F), while clustering of day two samples was according to temperature treatment (m and h). Values along branches indicate approximately unbiased p-values of Suzuki and Shimodaira [55] based on 1000 bootstrap replicates. Abbreviations: M - Mexico; F - Florida, 1 - 24 hours; 2 - 48 hours; m - mean temperature; h - high temperature.

To visualize the pattern of relationships among experimental treatments, a radial tree of hierarchically clustered gene expression profiles was constructed using 1,310 unigenes. Hierarchical clustering of gene expression data indicated that all three factors of time, location, and temperature had an effect on transcription profiles (Fig. 3). For 24 hour samples, the primary driver of gene expression profiles was geographic location, regardless of the treatment temperature at which they were raised. By 48 hours, however, the effect of thermal stress became the dominant factor, leading samples from Florida and Mexico to cluster together according to the treatment temperature experienced. Additionally, the cluster of high temperature samples after 48 hours of development was the most distant branch of the tree, implying a divergence from normal development to a signature profile resulting from a common response to thermal stress.

#### Principal component analysis

PCA supported the patterns detected by the hierarchical clustering. The first three principal components (PC) explained the majority (85%) of the variance inherent in the data, with PCs one and two already explaining over 76% (Fig. S1A). Plotting the treatments on the first two principal components showed that PC1 captured variation due to developmental time, while PC2 captured variation arising from geographic origin and water temperature (Fig. S1B).

#### Differential expression by developmental time

**Figure 4:**
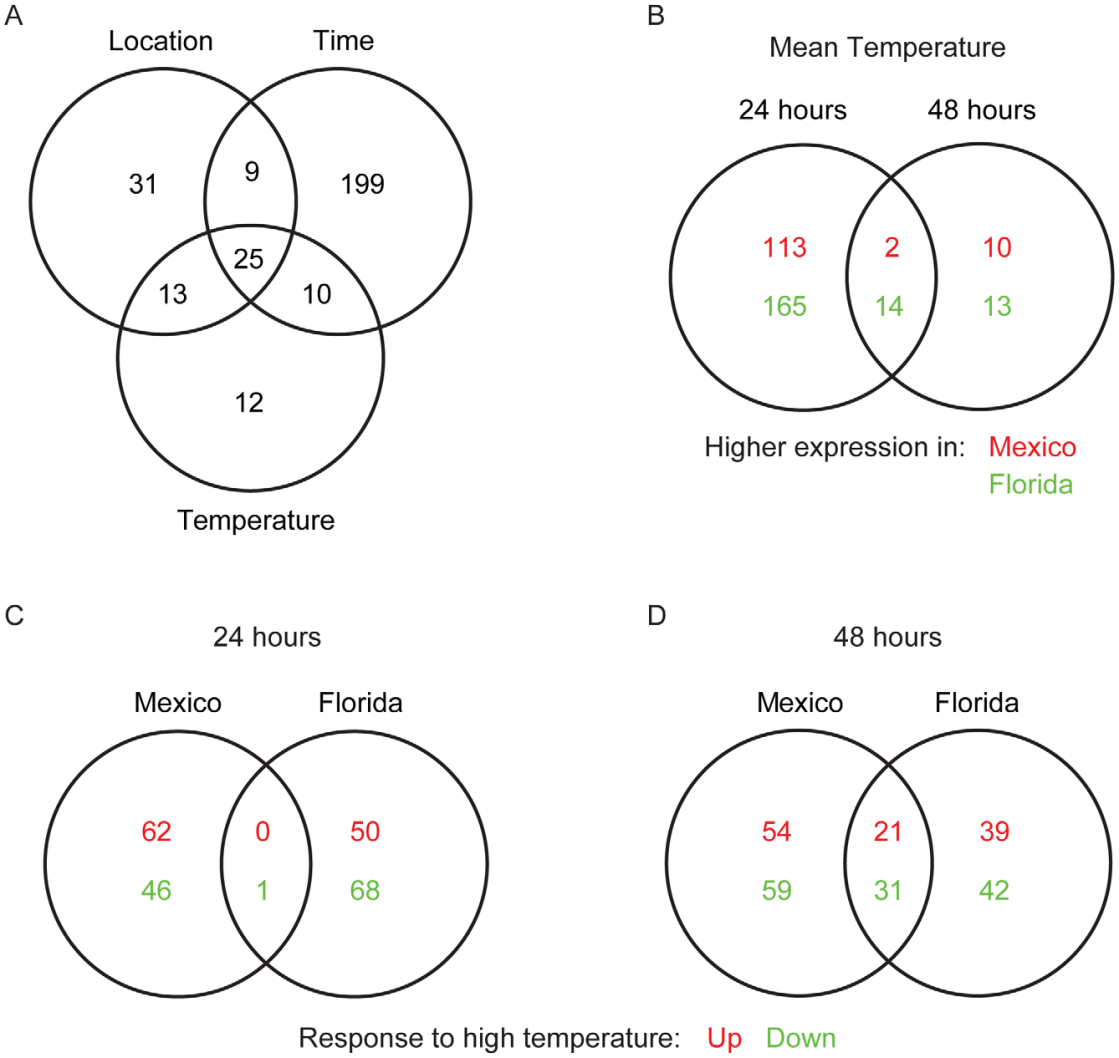
Overlap among differentially expressed genes in *M. faveolata* embryos from Florida and Mexico. (**A**) Differentially expressed genes (DEGs) responding to all three main effects of location, time and temperature were identified (note the large number of genes responding to the effect of developmental time). (**B**) When raised at control water temperatures the number of genes that were differentially expressed between the two locations was high at 24 hours, but dropped sharply by 48 hours. (**C**) DEGs responding to temperature differences show almost no overlap between locations at 24 hours (0 up, 1 down), but (**D**) considerable overlap by 48 hours. In **B**, upper and lower values indicate higher expression in Mexico relative to Florida. In **C** and **D**, upper and lower values indicate higher and lower expression levels respectively, under thermal stress conditions.

Consistent with Grasso *et al.* [56], the effect of developmental time drove the majority of changes in gene expression patterns overall (Fig. 4A; Time). The list of genes that were differentially expressed due to changes in developmental time was divided between sites to identify general and location-specific components (results not shown). While about 25% of DEGs responding to developmental time were shared between locations (n = 108), a large number of genes were differentially regulated in Mexico (n = 245) or Florida (n = 105) alone, providing a major component of the clear differentiation between locations observed in the hierarchical clustering (Fig. 3; left panel).

#### Differential expression by temperature treatment

DEGs responding to the temperature treatment were divided by location and developmental time to identify shared and location-specific components of stress response. Samples from both time points showed similar numbers of DEGs responding to the temperature treatment (Fig. 4C & 4D). Samples from 24 hours showed numerous DEGs responding to differences in temperature that are specific to Mexico (n = 108) and Florida (n = 118). However there was almost no overlap in genes responding to temperature between the sites. By 48 hours, the proportion of overlapping DEGs had risen (n = 52; 21% of all DEGs), while the number of location-specific DEGs remained similar to that observed at 24 hours (n = 113 in Mexico; n = 81 in Florida). DEGs responding to the temperature treatment that are common to both Mexico and Florida represent the component of stress response in *M. faveolata* that is shared between locations (Table S1). Temperature sensitive DEGs observed in only one of the two locations may represent location-specific strategies for coping with thermal stress (Table S2).

#### Differential expression by location under control temperatures

Genes that were differentially expressed between the locations under mean temperatures were examined to detect location-specific expression under non-stressful conditions (Fig. 4B). The greatest number of DEGs that varied by location at control temperature were observed in the 24 hour samples (n = 294). These are likely to represent variation in developmental processes between the locations that exist under normal conditions. By 48 hours, the number of genes that differed by location at control temperatures dropped dramatically to only 39 with very few transcripts (n = 16) carried over from 24 hours (Table S3).

### Functional Response

Gene Ontology (GO), and Kyoto Encyclopedia of Genes and Genomes (KEGG) annotation information was used to classify DEGs into functional groups when possible, based on the categories of Voolstra *et al.* [27]. Categories included: apoptosis; cell proliferation, growth and development; cytoskeleton and cell adhesion; DNA; electron transport and oxidative phosphorylation; metabolism; proteolysis and protein degradation; regulation of transcription; response to oxidative stress; response to stress; RNA; signaling; translation and protein biosynthesis; and transport.

Detailed investigation of gene function was performed on DEGs that represent the shared and location-specific components of the thermal stress response. Under the shared component of the temperature sensitive DEGs the functional category with the greatest number of transcripts was cell proliferation, growth and development (n = 5). Multiple transcripts (n≥3) were also involved in the processes of apoptosis; cytoskeleton and cell adhesion; DNA; proteolysis and protein degradation; and translation and protein biosynthesis. Location-specific components in Florida included cytoskeleton and cell adhesion (n = 7); proteolysis and protein degradation (n = 6); and translation and protein biosynthesis (n = 8). Other processes with multiple transcripts (n≥5) included apoptosis; cell proliferation, growth and development; cytoskeleton; metabolism; regulation of transcription; and transport. In Mexico, processes that were enriched for transcripts included translation and protein biosynthesis (n = 27), electron transport (n = 11), proteolysis and protein degradation (n = 7), transport (n = 7) and metabolism (n = 6). Enrichment of transcripts (n≥5) was also observed for the processes of cell proliferation, growth and development and response to oxidative stress.

## Discussion

The ability of corals to deal with changing climate conditions will depend upon the adaptive potential of the host as well as its symbionts. The results presented here expand upon our understanding of the effect of temperature stress on embryonic development in *M. faveolata* as described in Voolstra *et al.* [27], by extending the early developmental analysis from 12 to 24 hours, and adding a geographic component to the analysis. Variation in gene expression profiles of coral embryos was evident in the response to thermal stress at two locations within a species' range. We attributed these differences to variation in the host response alone because only aposymbiotic embryos were studied. The genetic response of adult corals is likely to differ somewhat from that of larvae due to symbiont interactions and changes in metabolic demands, however the observation of variation in these early life stages points toward the potential presence of adaptively significant variation upon which selection may be able to act. If this is the case, post-dispersal adult populations carry the signature of such early selection. While the limited biological replication in this study limits the extent to which they can be generalized, our results provide intriguing first insights into potential spatial variation in stress response of coral larvae in the absence of population differentiation.

A comparison of the genes that showed differential expression at the elevated treatment temperatures at 48 hours in the Voolstra *et al.* [27] study with those observed here reveals that multiple stress response genes including ferredoxin (AOSC403) calmodulin (AOSF573) and the proapoptotic caspase adapter protein (AOSF761) behaved consistently at high temperatures in both locations (Table S1). As did the histone proteins (AOSF1219 and AOSF622) and several other genes involved in cell proliferation, growth and development (AOSB596, AOSF1434, AOSF912), cytoskeleton and cell adhesion (AOSF1012, AOSF634), and translation and protein biosynthesis (AOSC1120, AOSF620, CAOO902). Peroxidasin (AOSF997) was also upregulated at both locations at 48 hours, but not significantly so in Florida. These genes represent a suite of transcripts that are likely to play an important role in the thermal stress response of corals from both locations.

There were also many stress response genes identified in Voolstra *et al.* [27] that differed between locations at the control temperatures in this study (Table S3). The stress response genes recombination repair protein 1 (AOSB392) and soma ferritin (CAON1101) showed differences between the two locations at control temperatures. The same was true for the cytoskeleton related gene dynein light chain roadblock - type 2 (AOSF651), and the system maintenance genes GTP-binding nuclear protein Ran -1 (AOSF912) and Nuclear hormone receptor - 6 (AOSB596), among several others involved in metabolism (CAON1380), signaling (AOSC876), DNA (AOSF622), and RNA modification (AOSF1077). Interestingly both populations showed differential expression of ribosomal proteins, although the specific protein homologs that showed differential regulation were not the same across locations, nor was the direction of change (up in Florida and down in Mexico). Variation in expression levels between sampling sites indicates location-specific molecular responses to environmental stress.

### Phenotypic Variation across Time and Space

The observation of malformed embryos provides a clear indication of the detrimental effect of elevated temperatures on larval development, especially in Florida. However, this is only one measureable phenotype of stress. The power of this study lies in the thirteen hundred transcripts that were measured simultaneously on the microarrays, revealing distinct patterns of gene expression by time, location and temperature (Fig. 3 & S1). Gene expression patterns indicated location-specific signatures of gene expression under average temperatures, as well as location-specific and conserved components of the response to elevated temperature as illustrated in the hierarchical tree (Fig. 3).

Gene expression levels measured by microarrays represent molecular phenotypes and will require additional research to identify the underlying genetic changes driving variation in expression levels [57]. Because variation in gene expression can reflect both heritable variation (i.e. changes in gene sequence or changes in transcript abundance due to differences in regulatory elements) and non heritable variation (arising from plasticity in response to environmental conditions, immune status, stress level, or physiological acclimatization to native habitats) only a subset of this variation can be expected to have true adaptive significance [58], [59]. However in studies where taxonomic divergence between groups has been taken into account, many transcripts exhibit changes in expression levels beyond what would be expected under genetic drift alone. This suggests that natural selection can play a role in changing expression levels in at least some loci [59]. Additionally, when heritability has been assessed, a large proportion of variation in gene expression is heritable and often involves changes in regulatory elements [57].

#### Development

The large number of transcripts that differ significantly in their expression level according to developmental time appears to be the primary factor driving the location-specific clustering observed in the hierarchical tree (Fig. 3; left panel). This result is in line with the results of Grasso *et al.* [56] where a large proportion of transcripts (21% of all surveyed) are differentially regulated with changing developmental time.

While studies of transcriptome profiles from larval invertebrates are limited, findings in polychaetes and ascidians suggest extensive variation in gene expression with development and onset of metamorphosis [60], [61]. Additionally, larval transcription profiles in abalone show that the expression of many transcripts are affected by interactions with exogenous cues such as settlement substrate [62], and in urchins, may be the target of extensive selective pressure [63].

#### Location

Distinct clustering of 24 hour embryos by location provides an indication that *M. faveolata* might show location-specific gene expression despite a lack of population differentiation [46].

Multiple taxa including killifish, yeast, *Drosophila* and humans have all shown high levels of intra-population variation in gene expression ranging from 18 to 83% [58], [64], [65], [66]. Evidence from the killifish, *Fundulus hereroclitus*, along a temperature cline suggests that variation in gene expression among populations is a positive function of within population variation. Following from neutral theory, divergence in gene expression patterns is expected to be proportional to taxonomic divergence between groups [59], and a large proportion of this variation must be due to genetic drift [67].

Considering the lack of divergence observed using neutral markers between the study sites in Florida and Mexico [46], it is unlikely that genetic drift would be strong enough to drive all of the observed transcriptional differences. Patterns of this sort have been observed in long lived trees, where low variation among populations according to neutral markers is accompanied by strong genetic clines in quantitative trait loci related to local climate adaptation [68], [69]. Alternatively, local acclimatization of mother colonies to annual mean temperature differences might affect larval development patterns [70].

Although thousands of larvae were analyzed in this study, increased replication over time and space will be necessary to provide confirmation of the patterns observed. This is a challenging task due to the vagaries of Caribbean coral spawning, difficult field conditions, and permitting constraints. Often, only some colonies/genets spawn on a given night. Consequently, the creation of genetically diverse larval pools with sufficient numbers of larvae to allow for a well replicated experimental design is difficult. We did not address the heritability of the putative location-specific stress response. Appropriate data would require rearing an F2 generation, a task that might be achievable in the future with further progress in coral husbandry [71]. Regardless of the mechanism, if substantiated, location-specific stress responses would have implications for both restoration planning and the study of coral evolution. This result supports the growing body of evidence indicating the potential for local variation in coral populations despite gene flow [7].

#### Temperature

The clustering of 48 hour samples into groups defined by stress treatment (Fig. 3) incorporates both a conserved response to thermal stress across locations, and location-specific components (Fig. 4C & 4D, Table S1 & S2). The observation of a common stress response only after 48 hours of development could be explained either by a true absence, or a failure to detect differential regulation in the 24 hour samples. Failure to detect early differential expression of some genes responding to temperature stress could be due either to changes in expression levels of a large number of genes related to development, or because sampling occurred long enough after the onset of the stress treatment that differential expression had ceased, as would be expected based on the results of Rodriguez-Lanetty [28], where HSPs were upregulated within 3 hours of exposure to thermal stress but not after 10 hours.

Embryogenesis of coral larvae is dominated by transcription of genes involved in cell replication and proliferation [56]. This dominance (see factor Time in Fig. 4A) may mask a functional response to thermal stress in early stages of development. Also, because stressful temperatures for this study were chosen based on local summer maxima and were intended to be permissive enough to allow for continued development without excessive mortality, the treatments may reflect expression of genes as a response to chronic high temperatures rather than a response to acute heat shock. This seems unlikely however, given the high mortality and number of misshapen embryos observed at the high temperatures.

The higher proportion of misshapen embryos could be a confounding factor for some of the location-specific DEGs unique to Florida at 48 hours, but not at 24 hours (Fig 4D &Table S2). Morphological anomalies were not apparent at 24 hours and we identified an even larger number of location-specific DEGs responding to temperature at this time point (Fig. 4C). Indeed, differential gene expression in response to temperature stress was likely the cause rather than the consequence of the abnormal morphologies observed at 48 hours. Nevertheless, Florida and Mexico samples from the high temperature treatments clustered tightly at 48 hours despite the higher proportion of visibly irregular larvae in Florida.

Previous studies of thermal stress tolerance in adult *M. faveolata* have observed expression of HSPs only after short term exposure to temperatures higher than 33°C [72], [73]. Our results provide additional evidence that the response of coral larvae to stress depends not only on the degree of stress, but the duration and timing of onset of the stress exposure [27].

### Functional Stress Response

The clear effect of thermal stress on embryonic transcription profiles supports the idea that the host plays an important role in coral thermotolerance [29], [74], [75], [76], though the fitness implications of this response are not yet known. Among the DEGs observed in response to thermal stress in both populations, a large proportion was categorized into the processes of cell proliferation, growth and development. Genes in this category were generally downregulated and it is hypothesized that this downregulation relates to the mechanisms underlying the abnormal morphology observed when larvae are grown at high temperatures (Table 1).

Other abundant categories were those of cytoskeleton and cell adhesion; electron transport and oxidative phosphorylation; metabolism; protein and proteolysis degradation; translation and protein biosynthesis; and transport. Genes related to translation and protein biosynthesis; proteolysis and protein degradation, electron transport and oxidative phosphorylation, were generally downregulated, while cytoskeleton and cell adhesion; metabolism; transport; response to stress; and response to oxidative stress genes were both up and downregulated. Together these results support previous models of stress response in corals and other taxa where an overall change in metabolic activity is associated with reduced protein biosynthesis, and oxidative phosphorylation [28], [36], [77]. Although there was substantial overlap in the functional categories of location-specific genes, many DEGs involved in the response to stress were unique to each location. This may represent locally differing strategies for dealing with thermal stress, but a thorough comparison of differences in molecular function across locations would require a larger, more representative microarray and a more complete functional annotation of cnidarian genes than is currently available.

#### Heat shock protein (hsp 90α) is downregulated

The conserved downregulation in both locations of the heat shock protein *hsp90α* (AOSC617; Fig. S2) was unexpected in light of its principle role as a molecular chaperone involved in response to thermal stress. This result is contrary to expectations based on previous studies that found HSPs were upregulated at high temperatures in adult *M. faveolata* [36], [72], 10 day old larval *A. millepora* [28], and other adult cnidarians [38]. The analysis of Voolstra *et al.* [27] did not show significant differential expression of *hsp90α* at 12 or 48 hours, but a slight upregulation was evident after 12 hours. Slight upregulation was also observed in the 24 hour sample here, but after 48 hours the pattern is strongly reversed such that the differential expression between mean and high temperature treatments over samples from both locations was statistically significant (Fig. S2).

Although the expression of HSPs in response to thermal stress appears to be universal [78], alternative forms of these proteins may be utilized in even closely related organisms [79], and a wide range of HSPs have been observed within the Scleractinia [38]. Since the microarray used here contains only homologs of *hsp90α* and *hsp97* it is possible that other HSPs were expressed but not detected.

Investigation of the cellular function of HSPs reveals that their role in the cell is not limited to protection from thermal stress however, and that they may be involved in a number of processes important to development including cell proliferation, cell cycle control, hormone receptor binding, microtubule formation and immune response [80], [81], [82]. Additionally, prolonged expression of HSPs 70 and 104 result in deleterious effects, representing a trade-off between thermotolerance and optimal growth and development in *Drosophila* and yeast [83], [84], [85].

The complex role of *hsp90α* suggests different functions for HSPs depending on the life stage of the organism and the environmental conditions experienced. Here, *hsp90α* was strongly downregulated in *M. faveolata* embryos that had been exposed to high temperatures for 46 to 48 hours, even though adult *M. faveolata* show an upregulation of *hsp90α* in response to stress after 10 days of exposure to high temperatures [36]. Similarly, 10-day old *A. millepora* larvae also showed upregulation of *hsp70*, *hsp90α* and *gp96* after up to 3 hours of exposure to thermal stress [28]. However, after 10 hours of exposure the difference was no longer significant, suggesting that the upregulation of HSPs may occur only immediately following exposure to stress. In light of this functional complexity and the strong downregulation of *hsp90α* observed in this experiment, it is proposed that *hsp90α* plays a developmental role in early stages of coral embryogenesis, and that upregulation of this transcript is no longer evident after several hours of chronic thermal stress.

#### Oxidative stress response

The response of corals to elevated temperatures is closely tied to their response to oxidative stress. Symbiotic cnidarians respond to high temperature with upregulation of numerous oxidative stress genes [36], [42], [86], [87]. Signals of the oxidative stress response can originate from the symbiotic algae as well as the animal host as a response to increased production of radical oxygen species in the mitochondria or chloroplasts during stress. Thus, the origin of the oxidative stress response in corals has been debated [88].

Our findings suggest that some of the oxidative stress genes that show differential regulation in response to temperature are indeed of animal origin. Two genes involved in the oxidative stress response; ferredoxin (AOSC403) and peroxidasin (AOSF997) were found to be significantly differentially regulated at high temperatures in both populations (Fig. S2). Other DEGs involved in the response to oxidative stress, included soma ferritin (CAON1101), malate dehydrogenase (AOSF1222), glutathione s-transferase *μ* (AOSF1447), and catalase (AOSF550). In contrast, the oxidative stress response of 10 day old *A. millepora* larvae was minimal after 3 hours but showed a slight, though statistically insignificant, increase in some transcripts after 10 hours [28]. This again suggests that developmental stage and/or timing of the onset of stress influences regulation of stress response genes. In light of the complexity of the oxidative stress response, it is difficult to say how differential regulation of these proteins will affect the cell, but downregulation of key enzymes, such as glutathione s-transferase *μ*, can change intracellular conditions leading to an overall enhancement of the oxidative stress response [89].

### Conclusions

Corals' ability to adapt to climate change depends upon their capacity to exploit functional genetic variation inherent to populations. In the current experiment, heritability of the detected variation was not assessed. While the genes that underlie differences among transcription profiles may differ in terms of sequence, copy number, or transcriptional regulators, observed differences could also be due to maternal effects and as such, gene expression profiles must be treated only as phenotypic variants. Further work is needed to establish the fitness consequences and heritability of observed changes in larval gene expression to determine the ecological and evolutionary significance this variation may have on the adaptive potential of corals' in the face of environmental change.

Our results add to a growing body of evidence that suggest considerable plasticity of coral gene expression profiles in the face of various stressors including high SSTs, predation, and turbidity [27], [36], [90], [91]. We provide support for location-specific signatures of gene expression in embryos of a reef-building coral from different parts of its geographic range. Moreover, we observe both location-specific and general components of stress response during later stages of development. Additional testing of the hypotheses presented in this work in combination with improved larval rearing techniques will help elucidate functional variation in natural coral populations and enhance conservation and restoration efforts by allowing managers to consider geographic variation in traits of importance to coral survival. Should further studies confirm the existence of ecotypes in corals in the face of gene-flow, ecological studies and management strategies would need to re-focus on micro-habitat characterization and conservation [69].

## Supporting Information

Table S1 Transcripts of M. faveolata embryos differentially expressed between the two treatment temperatures at both study locations. Up and downregulation is in relation to a common reference sample. Log2 fold change values in bold indicate significant results. Gene ID: NCBI EST database access number.
(0.04 MB XLS)

Table S2 Transcripts of M. faveolata embryos differentially expressed between the two treatment temperatures at only one study location. Up and downregulation is in relation to a common reference sample. Log2 fold change values presented for significant differences only. Gene ID: NCBI EST database access number.
(0.08 MB XLS)

Table S3 Transcripts of M. faveolata embryos differentially expressed between study locations at the control temperature. Up and downregulation is in relation to a common reference sample. Log2 fold change values presented for significant differences only. Gene ID: NCBI EST database access number.
(0.07 MB XLS)

Figure S1 Principal component (PC) analysis by treatment of transcription profiles from 24 and 48 hour M. faveolata larvae collected from Florida and Mexico illustrating the high explanatory power of the first two PCs (A). Plotting the treatments on the first two PCs shows that PC1 captures variation due to developmental time, while PC2 captures variation arising from both geographic origin in day one samples and temperature treatment in day two samples (B). Plotting against the 3rd PC (not shown) does not reveal any additional patterning. Symbols: M - Mexico (squares); F - Florida (circles), 1 - 24 hours (filled); 2 - 48 hours (open); m - mean temperature; h - high temperature.
(2.27 MB TIF)

Figure S2 Gene expression levels (log2 fold change) across all 8 treatments for stress response genes (Heat shock protein 90α; Transcription factor hes-1) and oxidative stress response genes (Ferredoxin; Peroxidasin) shared between Florida and Mexico. Abbreviations: M - Mexico; F - Florida; 1 - 24 hours; 2 - 48 hours; m - mean temperature; h - high temperature.
(6.64 MB TIF)
