# Supplementary figures and images for: Location-Specific Responses to Thermal Stress in Larvae of the Reef-Building Coral Montastraea faveolata
Source: PLoS One. 2010 Jun 23;5(6):e11221. doi: 10.1371/journal.pone.0011221 (PMC2890407; doi:10.1371/journal.pone.0011221)

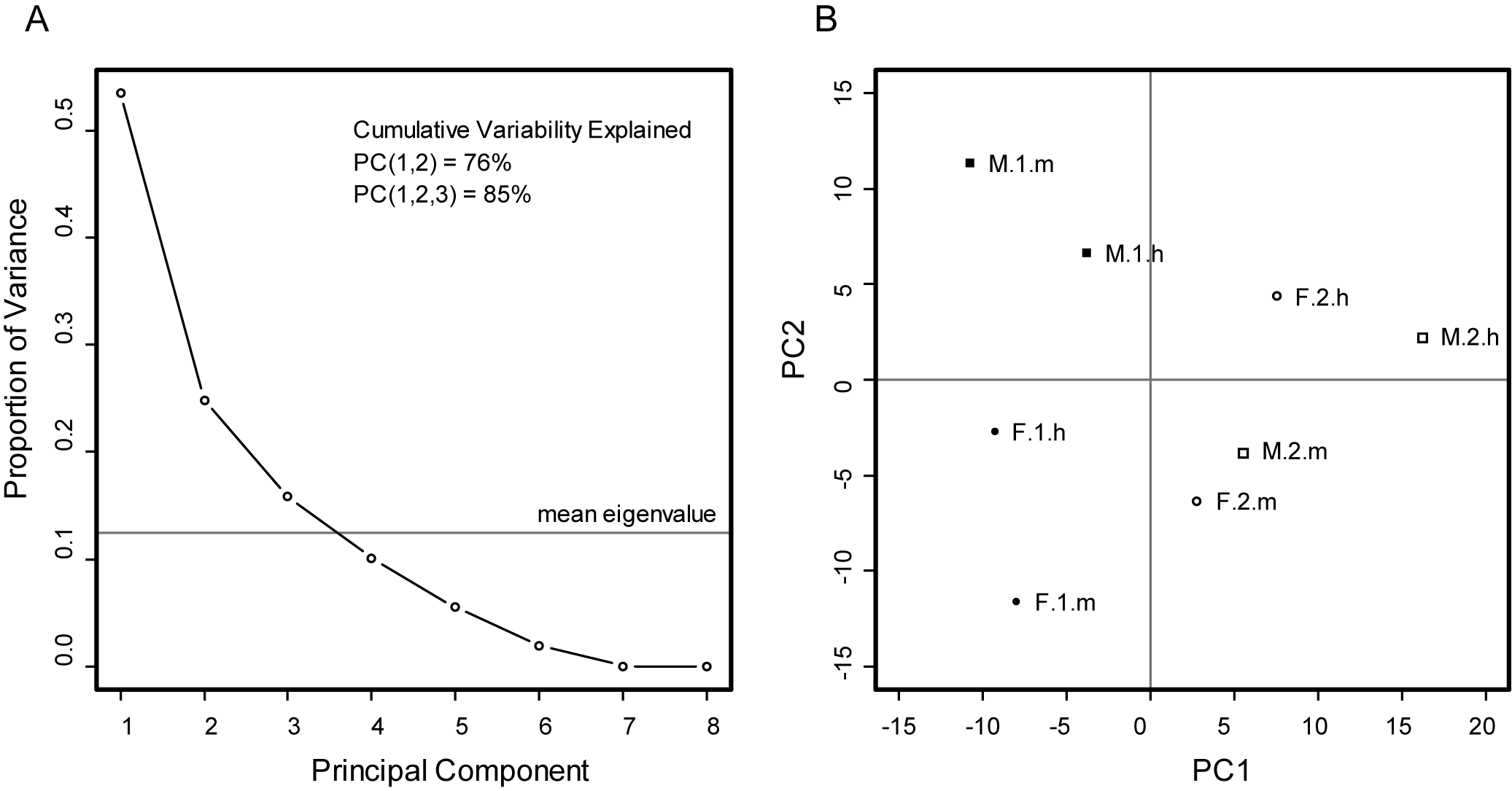

Supplement: Figure S1 — Principal component (PC) analysis by treatment of transcription profiles from 24 and 48 hour M. faveolata larvae collected from Florida and Mexico illustrating the high explanatory power of the first two PCs (A). Plotting the treatments on the first two PCs shows that PC1 captures variation due to developmental time, while PC2 captures variation arising from both geographic origin in day one samples and temperature treatment in day two samples (B). Plotting against the 3rd PC (not shown) does not reveal any additional patterning. Symbols: M - Mexico (squares); F - Florida (circles), 1 - 24 hours (filled); 2 - 48 hours (open); m - mean temperature; h - high temperature. (2.27 MB TIF) [file pone.0011221.s004.tif]

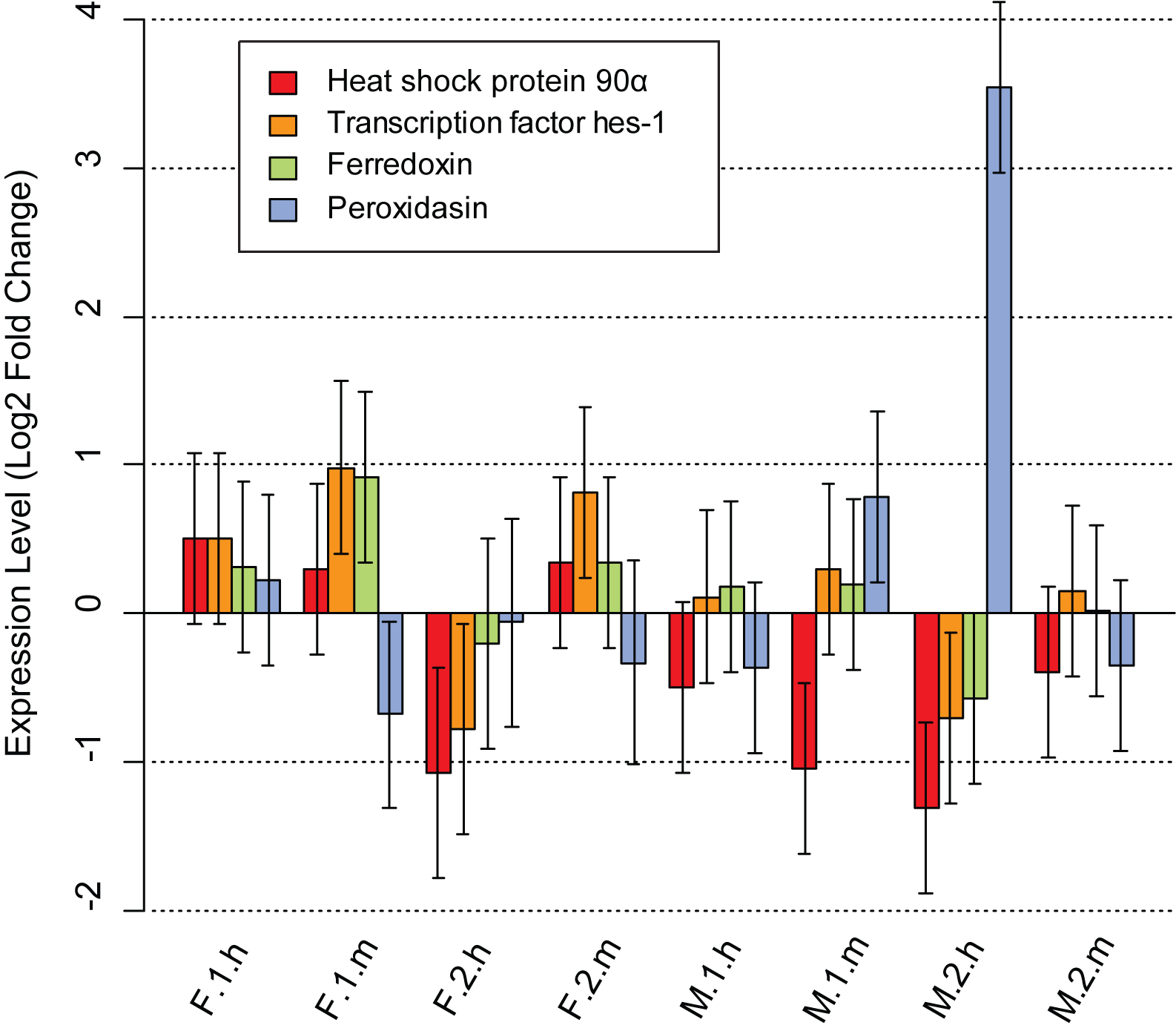

Supplement: Figure S2 — Gene expression levels (log2 fold change) across all 8 treatments for stress response genes (Heat shock protein 90α; Transcription factor hes-1) and oxidative stress response genes (Ferredoxin; Peroxidasin) shared between Florida and Mexico. Abbreviations: M - Mexico; F - Florida; 1 - 24 hours; 2 - 48 hours; m - mean temperature; h - high temperature. (6.64 MB TIF) [file pone.0011221.s005.tif]
